# Meaningful benefits: a framework to assess disease-modifying therapies in preclinical and early Alzheimer’s disease

**DOI:** 10.1186/s13195-022-00984-y

**Published:** 2022-04-19

**Authors:** Sheila Seleri Assunção, Reisa A. Sperling, Craig Ritchie, Diana R. Kerwin, Paul S. Aisen, Claire Lansdall, Alireza Atri, Jeffrey Cummings

**Affiliations:** 1 US Medical Affairs – Neuroscience, Genentech, A Member of the Roche Group, South San Francisco, CA USA; 2 Brigham and Women’s Hospital, Massachusetts General Hospital, Harvard Medical School, Boston, MA USA; 3 Edinburgh Dementia Prevention, Centre for Clinical Brain Sciences, University of Edinburgh, Scotland, UK; 4 Kerwin Medical Center, Dallas, TX USA; 5 Department of Neurology and Neurotherapeutics, University of Texas Southwestern Medical Center, Dallas, TX USA; 6 University of Southern California Alzheimer’s Therapeutic Research Institute, San Diego, CA USA; 7 F. Hoffmann-La Roche, Basel, Switzerland; 8 Banner Sun Health Research Institute, Sun City, AZ USA; 9 Center for Brain/Mind Medicine, Department of Neurology, Brigham and Women’s Hospital, Boston, MA USA; 10 Harvard Medical School, Boston, MA USA; 11 Chambers-Grundy Center for Transformative Neuroscience, Department of Brain Health, School of Integrated Health Sciences, University of Nevada, Las Vegas, NV USA

**Keywords:** Alzheimer’s disease, Clinical trials, Clinical meaningfulness, Meaningful benefit, Biomarkers, Preclinical, Mild cognitive impairment due to Alzheimer’s disease

## Abstract

**Background:**

The need for preventive therapies that interrupt the progression of Alzheimer’s disease (AD) before the onset of symptoms or when symptoms are emerging is urgent and has spurred the ongoing development of disease-modifying therapies (DMTs) in preclinical and early AD (mild cognitive impairment [MCI] to mild dementia). Assessing the meaningfulness of what are likely small initial treatment effects in these earlier stages of the AD patho-clinical disease continuum is a major challenge and warrants further consideration.

**Body:**

To accommodate a shift towards earlier intervention in AD, we propose meaningful benefits as a new umbrella concept that encapsulates the spectrum of potentially desirable outcomes that may be demonstrated in clinical trials and other studies across the AD continuum, with an emphasis on preclinical AD and early AD (i.e., MCI due to AD and mild AD dementia). The meaningful benefits framework applies to data collection, assessment, and communication across three dimensions: (1) multidimensional clinical outcome assessments (COAs) including not only core disease outcomes related to cognition and function but also patient- and caregiver-reported outcomes, health and economic outcomes, and neuropsychiatric symptoms; (2) complementary analyses that help contextualize and expand the understanding of COA-based assessments, such as number-needed-to-treat or time-to-event analyses; and (3) assessment of both cumulative benefit and predictive benefit, where early changes on cognitive, functional, or biomarker assessments predict longer-term clinical benefit.

**Conclusion:**

The concept of meaningful benefits emphasizes the importance of multidimensional reporting of clinical trial data while, conceptually, it advances our understanding of treatment effects in preclinical AD and mild cognitive impairment due to AD. We propose that such an approach will help bridge the gap between the emergence of DMTs and their clinical use, particularly now that a DMT is available for patients diagnosed with MCI due to AD and mild AD dementia.

## Background: the need for a multidimensional approach to assess meaningful benefits in clinical trials in earlier stages of the Alzheimer’s disease continuum

Alzheimer’s disease (AD) is a public health crisis [1–3] that is expected to worsen in the years ahead as the world’s population ages [4, 5]. However, the availability in the USA of the first AD therapy targeting the fundamental pathophysiology of the disease, along with the likelihood of other novel and potentially disease-modifying therapies (DMTs) to follow, are crucial steps toward addressing this substantial unmet need. In this context, it is imperative that we continue to expand our understanding of how best to measure and communicate the potential benefits of these AD medications as they start to enter clinical practice. Greater clarity on the relationships between biological effects (e.g., removal of amyloid plaques; effects on downstream biomarkers) and the benefit/risk profile of these medications, informed by biomarkers, will be transformative to AD care.

Interventions for AD must be associated with demonstrable benefit to patients, and these benefits must outweigh potential harms. Historically, drugs indicated for mild, moderate, or severe AD dementia have been approved based upon trials with co-primary endpoints that assess cognition as well as either functional or global clinical status; the two latter measures are intended to ensure treatment effects on cognition are *clinically meaningful* for patients and their caregivers [6]. More specifically, these medications are used for treatment of patients in the dementia phase of the illness with the intent of cognitive and functional improvement (or stabilization). Despite this explicit framework, translating the outcomes from clinical studies of these well-established symptomatic therapies to clinical practice and demonstrating the meaningfulness of changes has been difficult.

Challenges in assessing and communicating the meaningfulness of a treatment effect in AD are magnified for drugs designed to treat earlier, asymptomatic, or minimally symptomatic AD stages: (1) the available DMT and others currently under development are not expected to improve symptoms, but rather, to slow disease progression and mitigate clinical decline; (2) by definition, patients with preclinical AD are not experiencing symptoms and, therefore, would not be expected to demonstrate clinical improvement; (3) individuals in preclinical and early symptomatic phases of AD have limited or no clinical changes and drug-placebo differences are difficult to demonstrate; and (4) statistically significant differences between treatment and placebo arms are not necessarily clinically significant or meaningful without a persuasive definition of meaningful change on the selected trial endpoints. Pragmatically, the motivation for healthcare professionals, patients and their families, and payers to initiate and maintain therapy could be diminished if clear expectations for emerging DMTs are not established and if indicators of successful treatment are not communicated in a manner tailored to relevant AD stakeholders.

We propose *meaningful benefits* as a new umbrella term that describes the spectrum of potentially desirable outcomes that may be demonstrated in clinical trials across the AD continuum, with an emphasis on preclinical AD and early AD (i.e., mild cognitive impairment [MCI] due to AD and mild AD dementia). Our conceptualization of *meaningful benefits* extends the idea of *clinical meaningfulness* into populations without clinical symptoms. In addition, *meaningful benefits* may emerge downstream from intervention, in longer-term follow-up, and may be evident in clinical trials only via proxy measures or surrogate biomarkers and endpoints. Further, we base our recommendations about *meaningful benefits* on the reality that different stakeholders may desire different data outputs to interpret and contextualize the results of clinical trials, and ultimately, make informed decisions.

Thus, this proposal leverages the foundational components of clinical trials in AD (i.e., clinical outcome assessments [COAs]) while advocating for broader use and reporting of expanded outcomes beyond the “core” dementia phenomena of declining cognition and function [7], and encouraging the consistent application of complementary analyses. The former set of expanded outcomes encompasses measures of neuropsychiatric symptoms and socioeconomic burden, as well as patient- and caregiver-reported outcomes. The second component in this framework, complementary analyses, includes established and standardized statistical measures (e.g., number needed to treat [NNT]) that can be used to contextualize clinical trial results for a broader audience. Finally, meaningful benefits incorporate two additional, novel concepts believed to reflect key outcomes differentiating DMTs from symptomatic interventions: predictive benefit and cumulative benefits. *Predictive benefit* may be demonstrated if changes captured on a disease-relevant biomarker or on a core disease domain, such as cognition, predict longer-term clinical benefit, such as reduced clinical decline [8]. *Cumulative benefits* [9] reflect an accrual of effects over long-term therapy, such that the difference in outcome between those treated with placebo and those treated with drug increases over time. The recent approval of aducanumab under the accelerated pathway (i.e., that β-amyloid plaque removal is reasonably likely to predict clinical benefit) makes the concept of predictive benefit unquestionably relevant in AD drug development and clinical practice. Consistent reporting of AD clinical trial results in a manner that is mindful of the proposed meaningful benefits approach will serve to capture and communicate the anticipated benefits seen with DMTs in early phases of AD.

## The AD continuum, clinical staging, and regulatory guidelines

The concept of meaningful benefit takes on special relevance in relation to clinical development and regulatory pathways in preclinical and early AD. A brief overview of the staging criteria proposed by the US Food and Drug Administration (FDA) in their draft guidance for drug development in Early Alzheimer’s Disease: Developing Drugs for Treatment [10] and the AD continuum staging criteria suggested by the National Institute on Aging and Alzheimer’s Association (NIA-AA) [11] (Table 1) provide the context for a discussion of meaningful benefit in the early stages of AD.

**Table 1:** FDA-proposed stages for drug development vis-à-vis the preclinical AD, MCI due to AD, and AD dementia phases of AD proposed by NIA-AA

AD stage	Clinical/biomarker presentation	Cognitive and functional assessment	AD clinical continuum correlate
1	No cognitive impairment, including no subjective complaints, but AD pathology is present	No detectable abnormalities with sensitive neuropsychological measures	Preclinical
2	Transitional cognitive change from individual baseline, with cognition remaining within normal bounds and no functional impairment AD pathology is present	Detectable change on sensitive neuropsychological measures or subjective report of change	Preclinical
3	Subtle or more apparent objective cognitive impairment Impairment in ability to perform instrumental activities of daily living No loss of independence AD pathology is present	Detectable abnormalities on sensitive neuropsychological measures; mild but detectable functional impairments on sensitive measures	MCI due to AD
4	From 4 to 6, gradual progression on levels of cognitive impairment; impact on ability to perform basic activities of daily living and loss of independence AD pathology is present	Detectable abnormalities on COAs of cognition and function	Mild AD dementia
5			Moderate AD dementia
6			Severe AD dementia

*Abbreviations*: *AD* Alzheimer’s disease, *COA* clinical outcome assessment, *MCI* mild cognitive impairment

In its draft guidance, FDA noted that it is “highly desirable to intervene as early as possible in AD,” [10] making individuals in stages 1 and 2 (preclinical AD) and 3 (MCI due to AD) high-priority candidates for DMT trials. Congruent with the perspective outlined in this paper, the agency recognized the inherent difficulty in establishing any *clinically* significant impact of an intervention in trials including individuals in stages 1 and 2 because (1) by definition, these individuals have no clinical impairment to rescue; and (2) trials are likely to take several years to detect transition from asymptomatic to symptomatic disease.

Biomarkers of response to treatment in early AD are in a nascent period of development. At present, amyloid plaque reduction on amyloid positron emission tomography (PET) is accepted by the FDA as reasonably likely to predict clinical benefit and the basis for accelerated approval. There are many additional biomarkers in development (discussed below) that may ultimately be used to reflect successful therapeutic intervention and might be considered as surrogates for drug approval. The optimal biomarkers of different brain pathologies may differ across the AD continuum. Biomarkers may change without a corresponding clinical change and the value of a biomarker change may be better defined by what it predicts for later in the disease course. More data on the use of biomarkers across early stages of AD are being developed in trials and longitudinal cohorts.

## Investigating meaningful benefits across the AD continuum

One of the earliest attempts to define the tangible benefits, or “clinical meaningfulness,” of a drug for treatment of symptomatic AD was the 1990 FDA draft document by Leber [7]. This guideline stipulated dual-outcome measures for clinical trials: the use of a cognitive assessment in a clinical trial, such as the Alzheimer’s Disease Assessment Scale, Cognitive Subscale (ADAS-Cog) [12], ensured that an intervention had an effect on the “core phenomena of dementia”; drug–placebo differences on an additional global measure or functional measure established that treatment effects were clinically meaningful. These early efforts by FDA were important foundational concepts and remain influential, forming the core of clinical trial outcomes in stages 4, 5, and 6 (mild, moderate, and severe) AD dementia.

**Fig. 1:**
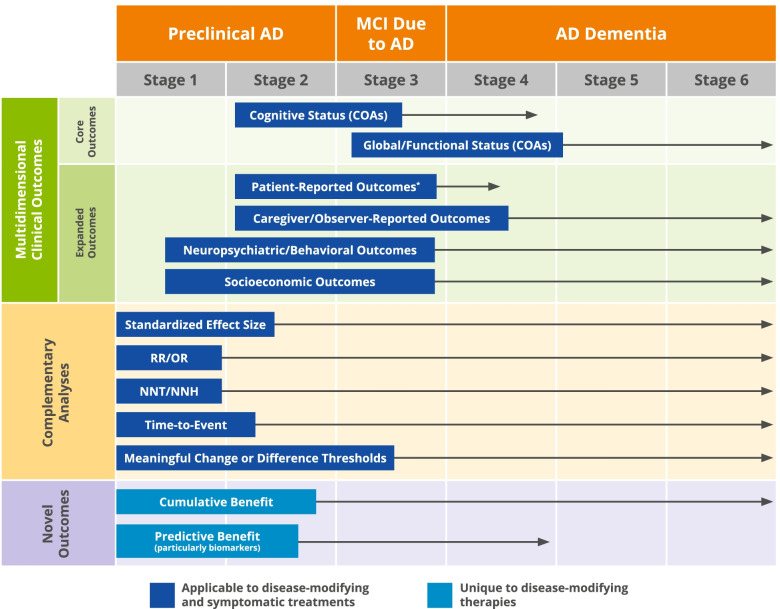
Meaningful benefits: a comprehensive spectrum of approaches across Alzheimer’s disease. Each assessment is introduced when it first becomes relevant in the disease continuum (boxes) and each arrow shows the length of time for which the assessment strategy remains relevant. The numbered stages refer to stage definitions introduced by the Food and Drug Administration. *The reliability of patient-reported outcomes may be compromised early in the expression of AD dementia; however, a number of different concepts can best be assessed with PROs, particularly when the concept being measured is best known to the patient or best measured from the patient’s perspective, such as subjective cognitive decline. Abbreviations: AD, Alzheimer’s disease; COA, clinical outcome assessment; DMT, disease-modifying therapy; MCI, mild cognitive impairment; NNH, number needed to harm; NNT, number needed to treat; OR, odds ratio; RR, relative risk

The expanded concept of meaningful benefits (Fig. 1) can advance drug development in the AD continuum as (1) it encourages a broad collection and comprehensive presentation of clinical trial data, which would ensure that a wide range of potential therapeutic dimensions of a DMT are considered, including some that have not been systematically investigated before; and (2) it conveys meaningfulness in multiple ways to resonate with various stakeholders, and to more clearly communicate the potential benefits of a given intervention.

## Multidimensional clinical outcomes assessed in Alzheimer’s disease trials

The clinical course of AD is characterized by a progressive deterioration in cognition that leads to impairment in daily functioning, loss of independence and, consequently, increased caregiver burden. Preserving cognitive and functional domains—or slowing their deterioration—are central goals in AD clinical trials and treatment. However, a valid assessment of the benefit of any intervention for AD must reflect and encompass a diverse set of outcomes. A drug’s effect on AD can be assessed using a variety of COAs; for example, a recent review by Webster and colleagues [13] identified 81 different COAs of cognition, activities of daily living, neuropsychiatric symptoms, quality of life, global well-being, and biomarkers used in trials of mild to moderate AD dementia. Beginning with core outcomes related to cognition and function, we review how an assessment of meaningful benefits should address outcomes across a full range of domains and measures.

### Core outcomes

As studies in AD have moved toward assessing patients in earlier stages along the disease continuum, established COAs to measure cognition or functioning have proven to be less sensitive in detecting changes over time in asymptomatic or minimally symptomatic populations [14, 15], demonstrating, for example, ceiling effects in MCI and mild AD dementia [6] or little change over the course of short trials in preclinical or early AD [16]. Recognizing the limitations of existing AD assessments, researchers and clinicians have modified and refined existing tools and have developed novel instruments that are sufficiently sensitive to detect more subtle changes over time in preclinical AD and MCI due to AD (Table 2) [12, 16–42].

**Table 2:** Outcome measures in early phases of the AD continuum^a^

Domain	Preclinical AD^b^	MCI due to AD
Cognition	**Preclinical Alzheimer Cognitive Composite (PACC)** • Composite cognitive measure that assesses episodic memory, timed executive function, and global cognition • In several studies, has reliably identified cognitive decline in individuals with preclinical AD over a 2- to 3-year observation period [17] • Primary endpoint in first interventional trial in cognitively normal individuals identified as “at-risk” for progression to AD dementia—the Anti-Amyloid Treatment in Asymptomatic Alzheimer’s (A4) study [18] **Alzheimer’s Prevention Initiative Composite Cognitive Test (APCC)** • Composite instrument of word recall, naming, praxis, orientation, and abstract reasoning [19] • Primary outcome measure in Alzheimer’s Prevention Initiative (API) [19] • Sensitivity has been independently confirmed in a cohort of cognitively normal older adults who progressed to late-onset AD [20]	**Alzheimer’s Disease Assessment Scale, Cognitive Subscale (ADAS-Cog)** • 11 subtests related to memory, praxis, and language [12] • Standard tool in pivotal clinical trials to detect therapeutic efficacy in cognition [21] • Best for patients with symptomatic AD; although often used in earlier AD, ceiling effects may limit its utility unless the assessment is modified (e.g., adding one or more tests) [21, 22] **Neuropsychological Test Battery (NTB)** • Uses widely available tests with known reliability and validity to overcome some of the limitations of the ADAS-Cog • Assesses 5 cognitive domains: attention, language, memory, spatial, and executive function • High degree of reliability for characterizing individuals with mild to moderate AD [23–25] • More sensitive to change in mild AD dementia than the ADAS-Cog [23–25]
Global/composite	None	**Clinical Dementia Rating (CDR) – GS or SB** • Clinician rating scale widely used in clinical trials in MCI due to AD and mild AD dementia • Can yield global score (CDR-GS) or sum of boxes (CDR-SB) • CDR-GS supports trial eligibility and staging: score of 0.5 corresponds to MCI; score of 1 corresponds to mild dementia [26] • CDR-SB is often the primary outcome; has both cognitive (e.g., memory, orientation, judgment, and problem solving) and functional (community affairs, home and hobbies, personal care) aspects and serves as a composite measure **Alzheimer’s Disease Composite Score (ADCOMS)** • Composite scoring approach designed as outcome measure for trials in patients with MCI due to AD and mild AD dementia [27] • Based on weighted scores from 4 ADAS-Cog subscale items, 2 Mini-Mental State Examination (MMSE) items, and all 6 CDR items • Demonstrated improved sensitivity over individual scales to detect clinical decline in people with amnestic MCI and those individuals with mild AD dementia • Also detected treatment effects associated with the use of cholinesterase inhibitors in these populations **Integrated Alzheimer’s Disease Rating Scale (iADRS)** • Combines scores from the ADAS-Cog and the Alzheimer’s Disease Cooperative Study—Instrumental Activities of Daily Living (ADCS-iADL) • Across all phase 3 trials of the anti-amyloid treatment, solanezumab, in mild AD dementia, the iADRS differentiated between active treatment and placebo [28]
Function	**Emerging options** • Performance-based assessment of functional capacity as demonstrated by: o A performance-based assessment of everyday function [29] like the University of California San Diego Performance-Based Skills Assessment (UPSA), or o Navigating an interactive voice response system, as in the Harvard Automated Phone Task, [30] or o Demonstrating skills in a virtual setting, such as the Virtual Reality Functional Capacity Assessment Tool (VRFCAT) [31] • Brief performance measure of financial skills, such as with the Financial Capacity Instrument [32] • Informant- and/or patient-reported ratings of everyday function, such as o The Everyday Cognition (ECog) scale [33] o The Cognitive Function Index (CFI) [34, 35] o The ADCS-ADL Prevention Instrument (ADCS ADL-PI) [36, 37]	**ADCS-ADL-MCI Scale** • Widely used endpoint in clinical trials [38] • Inventory of ADL elements rated based on the extent of assistance the individual needs with each activity (e.g., going shopping or keeping appointments or meetings) [38] • Successfully distinguishes those with MCI from those with unimpaired function [39] **Amsterdam IADL Questionnaire** • Study partner report about the individual’s ability to perform a range of everyday activities, including cooking, finances, and everyday technology use [40] • Correlates longitudinally with cognitive decline [41] • Detects difficulties with IADLs in preclinical AD compared to healthy controls [42]

*Abbreviations*: *AD* Alzheimer’s disease, *MCI* mild cognitive impairment

^a^A more comprehensive review of a range of composite batteries developed for secondary prevention trials in AD, as well as their strengths and weaknesses, was recently provided by Schneider and Goldberg [16]

^b^FDA has suggested an integrated scale that adequately and meaningfully assesses both daily function and cognitive effects. This scale may be acceptable as a single primary efficacy outcome in trials of preclinical AD

FDA guidance on the development of drugs in early AD has been an important catalyst to the development, validation, and application of novel COAs in preclinical and early AD. For example, FDA has noted that in stage 3 AD, a composite scale that adequately assesses daily function and cognitive effects may be acceptable as a single primary efficacy outcome measure [10]. The Clinical Dementia Rating sum of boxes (CDR-SB) is the most commonly used primary endpoint in phase 3 clinical studies in this population; some phase 2 studies, however, have used other composite analyses and tools such as the Alzheimer’s Disease Composite Score (ADCOMS) and Integrated Alzheimer’s Disease Rating Scale (iADRS).

In stage 2 AD (i.e., preclinical with transitional cognitive change), FDA has suggested that a persuasive effect on one or preferably more sensitive measures of neuropsychological function along with effects on characteristic pathophysiologic changes of AD may suffice for drug approval. In this context, most trials in this stage include continuous measures as the primary endpoint, such as the Preclinical Alzheimer Cognitive Composite (PACC) or the Alzheimer’s Prevention Initiative Composite Cognitive Test (APCC) (Table 2) [12, 16–42]. Recent studies suggest that early change on the PACC is associated with subtle functional decline and is predictive of future functional impairment [43]. FDA indicated that the use of a time-to-event (TTE) survival analysis—such as time to progress from stage 2 to stage 3—can be an acceptable efficacy measure in early AD trials, and such TTE endpoints have been included in some ongoing trials as either primary or secondary measures [44]. One point of discussion regarding TTE is the lack of broad consensus on how to define a meaningful event in the earlier stages of the AD continuum—especially one that is considered consistently meaningful, despite heterogeneity in the population.

In describing trials in stage 1 AD, FDA has stated that it may suffice to demonstrate an effect on pathophysiological changes of AD, as shown by an effect on one or more biomarkers—assuming that the biomarker effect is reasonably likely to predict clinical benefit in the future. Designing trials for preclinical AD intervention is in its relative infancy; neither change on a specific biomarker nor change on any of the above-mentioned cognitive composite batteries is yet to be linked to meaningful outcomes such as a delay in onset of MCI or dementia or a delay in functional decline [15]. FDA’s approval of aducanumab for use in patients with early AD may motivate the use of surrogate markers in preclinical AD studies.

### Expanded outcomes

Meaningful benefits in preclinical and early AD should include patient- and caregiver-reported outcomes, health and economic outcomes, and neuropsychiatric symptoms.

#### Patient- and caregiver-reported outcomes

The benefits reported by patients or individuals at risk of developing symptoms as a result of receiving an intervention reflect an important dimension of intervention effectiveness [45]. Perceived patient benefits may be systematically collected via patient-reported outcome (PRO) measures; PROs are defined as any report of the status of a patient’s health condition that comes directly from the patient, without interpretation of the patient’s response by a clinician or anyone else [46]. Preservation of everyday functioning, maintaining relationships and social connections, enjoying life, preserving a sense of identity, and alleviating symptoms are the qualities consistently identified by cognitively normal older individuals as desirable outcomes of a hypothetical DMT and are target outcomes to be captured by PROs [46, 47]. A recent review by the Alzheimer’s Disease Patient and Caregiver Engagement initiative demonstrated that some of the most consequential symptoms and effects of AD as identified by patients and their care partners are not adequately captured by widely used COAs, indicating the need for companion tools to fully capture concepts of interest for patients and care partners [48].

Validated PROs are needed in AD trials and some are under development [46, 49, 50]. The Patient-Reported Outcome Consortium’s Cognition Working Group had led efforts to develop a novel, self-reported outcome measure in persons with MCI due to AD, emphasizing two functional domains: (1) complex activities of daily living (e.g., handling personal finances and meal preparation) and (2) interpersonal functioning (e.g., conversational skill or comprehension of written material) [49]. However, concerns that the characteristic loss of insight among patients with MCI due to AD could impact the reliability of a PRO in this target population led the working group to refocus on qualification of a performance-based measure assessing the ability to perform instrumental activities of daily living [51].

Although PROs have limited use in AD dementia due to patients’ loss of insight and/or memory loss, they could be particularly important in the preclinical and early AD stages (stages 1–3), when insight is preserved and changes may be too subtle to be reliably observed by others (e.g., clinicians, friends, or family members). More studies are needed to determine the utility of such measures in trials in stages 1–3.

Finally, regulatory agencies encourage PRO assessments in clinical studies [52]. For the collection of patient experience data, FDA recommends direct reports from patients, unless they are unable to report reliably on the concept of interest [53]. When collection of direct patient experience is limited, valuable but distinct information may still be obtained from informants, such as family members and/or caregivers [53].

#### Health and economic outcomes

Health and economic outcomes represent another expanded means of assessing meaningful benefit. AD is one of the costliest diseases to society; expenses include direct costs (e.g., medical and residential care payments), indirect costs (e.g., the unpaid work of informal caregivers), and intangible costs (e.g., diminished quality of life for patients and caregivers; caregiver burden [54]. Not only are these impacts substantial, accruing in greater amount over the course of symptomatic AD, but most of these costs and burdens begin even in the years before the onset of clinical manifestations—that is, when a person is experiencing MCI or early symptomatic dementia [54]. Different stakeholders—and indeed, even different payers (e.g., private payers, employers, Medicare)—may value different health and economic outcomes (e.g., placing more or less value on retained worker productivity). Significant data must still be accrued to enable dialogue around the benefit captured by different assessments. Major health and economic goals associated with treatment in early AD—some of which may not be fully evident until later stages of the disease continuum—include reduced formal and informal resource utilization (Table 3) [55–58] as well as retained patient autonomy. These outcomes may translate into reduced caregiver burden and, ultimately, reduced institutionalization or prolonged time to nursing home placement [59].

**Table 3:** Health and economic outcomes and neuropsychiatric symptom measures for early AD

Domain	Potential Measure
**Health and economic outcomes**	**Resource Utilization in Dementia (RUD) Questionnaire** • Structured interview with study partner to obtain information about patient and caregiver, including [55] o Healthcare resource utilization o Work status o Living accommodations o Level of formal and informal care attributable to AD, including caregiving time spent assisting patient’s instrumental ADLs or basic ADLs • Emerging evidence leveraging the RUD indicates that early AD, including MCI, poses a financial burden to the patient, caregiver, and society [56]
**Neuropsychiatric symptoms**	**Neuropsychiatric Inventory** **[**57**]** • 12-item informant-based interview about delusions, hallucinations, anxiety, depression, agitation/aggression, euphoria, disinhibition, irritability/lability, apathy, aberrant motor activity, night-time behavioral disturbances, and appetite/eating abnormalities • Widely accepted measure of neuropsychiatric symptoms in dementia **Mild Cognitive Behavioral Impairment Checklist (MBI-C)** **[**58**]** • Specifically developed as an MBI case ascertainment instrument, which also allows for the monitoring of MBI symptoms over time • 34-item instrument for completion by patient, close informant, or clinician • Assesses 5 domains of (1) decreased motivation; (2) emotional dysregulation; (3) impulse dyscontrol; (4) social inappropriateness; and (5) abnormal perception or thought content

#### Neuropsychiatric symptoms in AD

Neuropsychiatric symptoms (e.g., depression, agitation, psychosis, apathy, anxiety, irritability, and social withdrawal) are well-known manifestations of AD [60]. The emergence of these symptoms late in life may reflect the development of mild behavioral impairment (MBI), an “at-risk state” for cognitive decline and dementia that may arise ahead of or in parallel with MCI [60, 61]. The effect of neuropsychiatric symptoms in the AD continuum can be severe, as they are associated with reduced quality of life, earlier institutionalization, faster disease progression, increased caregiver stress, and greater overall costs of care [60, 62]. Thus, neuropsychiatric symptoms in AD may be present and detected early, and both their initial expression and their long-term course may serve as important markers of meaningful therapeutic benefits. Understanding of MBI and early manifestations of these symptoms is less robust earlier in the AD continuum compared with understanding of these same phenomena in later disease stages. However, existing tools and emerging ones (Table 3) [55–58] may help guide our current assessment of incident and prevalent neuropsychiatric symptoms in early AD [63]. Reduction in the severity or emergence of neuropsychiatric symptoms is a key measure of meaningful benefit.

## Complementary analyses: expanding assessments and reframing clinical trial data to communicate the full spectrum of potential benefit from DMTs

Certain elements of clinical trials, such as co-primary endpoints, may be of particular relevance only to select audiences (e.g., regulators and clinical trialists). However, a diverse set of stakeholders is involved in the care of individuals with AD and the payment for care, and each stakeholder may require analyses that differ from outcomes used in trials. Table 4 summarizes complementary analyses that may further contextualize trial data [10, 17, 42, 53, 64–72]. With exception of effect size, the other analyses require the definition of an event of interest. AD stakeholders may assign different values to certain events, and some events may be more reliably assessed than others. Furthermore, defining “an event” that is relevant for all stakeholders is a challenge in a disease as heterogenous as AD. Thus, research is ongoing to understand how to best define and assess discrete events in preclinical and early AD trials.

**Table 4:** Analyses to convey clinical trials results in non-trial settings

Analysis	Interpretation	Potential utility/application
**Standardized effect size (e.g., Cohen’s** ***d*)**	• For a comparison of group means, Cohen’s *d* large effect size: ≥0.8; medium effect size 0.5–0.8; small effect size: 0.2–0.5 • Cohen’s *d* can be applied to any continuous measure, including those used in preclinical AD and MCI due to AD [42, 64]	• Effect sizes can provide an interpretable index of the direction and magnitude of the effect of an intervention [65–67] • Because effect size estimates enable some control of variability, they also allow for some level of comparison across similar studies [65–68]
**Relative risk (RR)/odds ratio (OR)**	• Complement standard effect sizes such as Cohen’s *d* [65–67] • Useful for estimating effect sizes from categorical measures, such as “improved” versus “not improved” or “converted to MCI” or “did not convert to MCI” [66, 69]	• Both RR and OR are ways in which clinicians often generally consider treatment effects [66]
**Number needed to treat (NNT)/number needed to harm (NNH)**	• A high NNT indicates a less effective treatment [70]: interventions with an NNT in the single or low double digits are generally considered effective, although an NNT in the lower hundreds may also be considered useful, depending on the significance of the outcome, such as preventing death [71] • NNH provides similar index for the occurrence of one or more specified adverse events	• NNT, which is related to absolute risk reduction, may be the effect size estimate that best reflects clinical significance for binary outcomes such as success or failure [66]
**Time-to-event (TTE)**	• Versatile analytical method also known as survival analysis [17] • Refers to a set of methods for analyzing the length of time until the occurrence of a well-defined endpoint of interest [17] • In preclinical and early AD trials, an outcome of great interest is conversion from one stage (e.g., preclinical AD) to the next (MCI due to AD) on the AD continuum [17]	• Determining what is considered a meaningful event can be challenging, especially in early AD. In addition, operationalizing transitions may be burdensome given the subtle differences between AD stages; nonetheless, TTE analyses may provide useful information as part of a broader evaluation of effect • Recognized as endpoint option by both FDA and EMA [10]
**Meaningful change or difference threshold**	• Reflects the level of score change(s) on a COA that is perceived to be meaningful in the target patient population • There are two main approaches: o Clinically meaningful change thresholds for individual patients (within-patient approach, recommended by the FDA) [53] o Clinically important difference thresholds applied at the group level (between-groups approach) [72] • The aim is to ensure that the observed treatment benefit as measured by the COA is meaningful to a patient	• Responder analyses convey the proportion of patients who meaningfully respond to treatment (i.e., achieve or exceed the within-patient meaningful change threshold) • In the context of a progressive disease like AD, a progressor analysis may be more appropriate (i.e., define meaningful progression on the COA), to demonstrate the proportion of patients who meaningfully progress on treatment vs placebo • Exceeding a threshold for MCID between groups can support the meaningfulness of a statistically significant treatment effect at the group level

The contents of this table are representative of complementary analyses, and do not reflect a comprehensive list

*Abbreviations*: *AD* Alzheimer’s disease, *COA* clinical outcome assessment, *EMA* European Medicines Agency, *FDA* US Food and Drug Administration, *MCI* mild cognitive impairment, *MCID* minimum clinically important difference

Effects sizes, a means of assessing benefit and comparing interventions, have been widely used in studying treatment but have had limited application in AD. For example, cholinesterase inhibitors are known to produce small to moderate effect sizes in clinical trials on both continuous and ordinal measures of cognition and global well-being [73]. Small to medium effects on cognition and global scales have been documented in moderate to severe AD dementia treated by memantine [74]. Effect sizes for long-term real-world treatment of AD dementia with cholinesterase inhibitors with and without add-on therapy with memantine have also been reported [68, 75]. “Effect sizes” for categorical measures may be best expressed in terms of relative risk (RR) and odds ratio (OR): when comparing a treatment with placebo, an RR or OR of 1 indicates that outcomes did not differ between the two groups, whereas an RR or OR > 1 indicates an increased probability of the event in the treatment group. For example, an RR of 5 indicates that the treatment group had a fivefold greater probability of showing improvement than the placebo group. Fields such as oncology have embraced RR and OR for estimating the ability of a treatment to prevent or delay progression or mortality.

The NNT with a cholinesterase inhibitor has been calculated as ranging from 4 to 14, whereas the number needed to harm (NNH) for the most common adverse reactions with this class of agents is between 6 and 20 [64], indicating a relatively favorable benefit/harm ratio. NNT/NNH ratios will be important for DMTs, especially those that may produce amyloid-related imaging abnormalities, and particularly as NNT and NNH calculations are often used to compare interventions and to guide reimbursement decisions [76]. NNT can be combined with TTE analyses to determine the NNT to prevent one patient from progressing from one stage to the next.

Meaningful change or difference thresholds are often used to aid interpretation of COAs [77] by defining a score change (or range of score changes) beyond which a patient or group of patients may be considered to have “responded” meaningfully to treatment [78]. Thresholds should be established a priori using gold-standard methodology and informed by patient need [79]; they can be applied to clinical trial data to support the interpretation of results at the individual within-patient level (FDA-recommended approach; e.g., responder analyses) or at the group level (e.g., minimal clinically important difference) [80].

Ultimately, these complementary analyses can be applied to clinical trial data and may help convey the results of ongoing DMT trials in AD to different audiences and support cross-study comparison (see Keefe et al. [65] for a more comprehensive assessment of the role and utility of different measures for different audiences).

## Cumulative and predictive benefits with DMTs

**Fig. 2:**
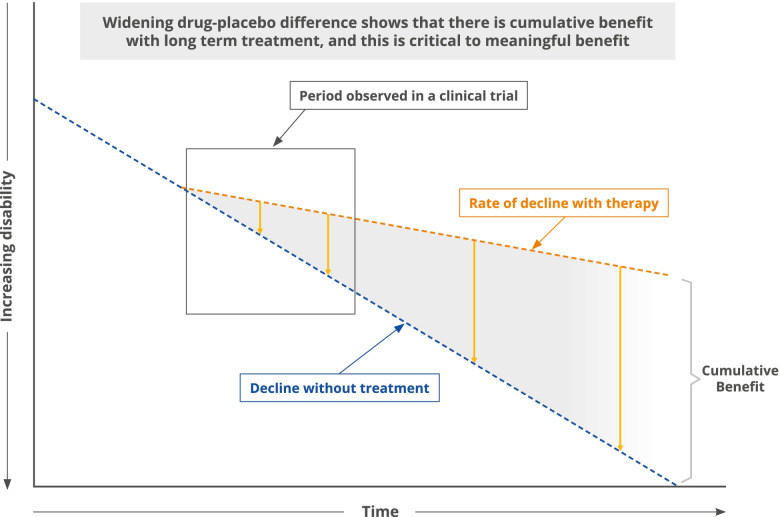
Theoretical rate of decline with DMTs. Model showing the theoretical rates of decline with disease-modifying treatment and without. There is an increasing drug–placebo difference over time with cumulative benefit of long-term therapy. Adapted from Cummings & Zhong [81]

Finally, we anticipate both *cumulative* and *predictive* benefits seen with DMTs to be essential components of the meaningful benefits associated with these drugs in the earliest stages of AD. A unique feature of DMTs compared with symptomatic AD treatments is that DMTs slow cognitive and functional decline, manifested by a change in the slope of decline and an increasing drug–placebo difference over time (Fig. 2) [81]. The benefit of DMTs is time-dependent and the value of intervention with these therapies is anticipated to increase as the patient’s duration of therapy increases [6]. As a result, included within the meaningful benefits of DMTs is the *cumulative benefit* of long-term therapy, a unique aspect to DMTs when compared with symptomatic therapies [82–84]. For example, studies have demonstrated that disease-modifying immunotherapies for multiple sclerosis reduce disability accrual over the short term of 1 to 3 years [85], and more recent real-world evidence has demonstrated long-term cumulative benefit over years to decades [83, 86]. Studies of DMTs in patients with multiple sclerosis have shown benefits of starting DMTs earlier in the disease course compared with later, including improvement in mortality and reduced disability over the longer term [87, 88]. Understanding how to measure the cumulative effects of AD DMTs is crucial to characterizing their full benefit; as more AD DMTs become available, data from observations beyond the trial period will allow greater insight into long-term outcomes.

From a regulatory perspective, COAs continue to be the gold standard for demonstrating efficacy of a medication. In several disease areas, however, certain biomarkers qualify as surrogate endpoints because treatment effects on the biomarkers predict clinical benefit. This advancement has contributed to the more expeditious development of new therapies [89, 90]. The importance of these biomarkers is observed in clinical practice, where they are used to inform the need for and success of treatment, including both well-validated biomarkers (e.g., HbA1_c_ as a predictor of diabetic microvascular complications, or blood pressure as a predictor of primary and secondary cardiovascular events) as well as ones that are less well validated but still provide important information (e.g., RNA copy number for clinical monitoring of antiviral therapy in patients with HIV) [91, 92].

Thus, the second novel concept to be integrated into the meaningful benefit framework is *predictive benefit*. In AD, this concept may be indexed by early changes on cognitive or functional assessments that predict long-term outcomes [6], although it is more commonly expected that a change or an impact on an underlying biomarker might serve as a surrogate that predicts eventual clinical benefits of an AD DMT. As biomarker knowledge has grown, many drugs in different therapeutic areas have been approved—including aducanumab for the treatment of AD—on the basis of changes in surrogate biomarkers considered “reasonably likely” to predict clinical benefit [93, 94]. Biomarkers used in this way are not fully validated surrogates for clinical benefit, and approval using this approach is coupled with the requirement to confirm beneficial clinical effects with additional studies. Knowledge about AD is growing rapidly and surrogate markers or more biomarkers “reasonably likely” to predict benefit are anticipated to emerge. Many of these biomarkers (Table 5) have been included in long-term trials [95] embedded in large collaborations, such as the Alzheimer’s Disease Neuroimaging Initiative [95, 96], or collected in industry-sponsored clinical trials.

**Table 5:** AD biomarkers used in assessing disease state or prognosis: potential surrogate markers

Source	Biomarkers
Cerebrospinal fluid	Aβ42 (alone or when measured as a ratio with Aβ40, total tau, or p-tau)
	Total tau
	Phosphorylated-tau (p-tau; alone or when measured as a ratio with Aβ42)
	β-site amyloid precursor protein cleaving enzyme 1 (BACE1)
	Triggering receptor expressed on myeloid cells 2 (TREM2)
	YKL-40
	Neurogranin
	Synaptosome-associated protein 25 (SNAP-25)
	Synaptotagmin
	Visinin-like protein 1 (VILIP-1)
	Neurofilament light (NfL)
Blood	p-tau 181; p-tau 217
	Aβ42/Aβ40 ratio
	Neurofilament light (NfL)
Imaging	Amyloid PET
	Tau PET
	MRI atrophy
	FDG PET hypometabolism

*Abbreviations*: *AD* Alzheimer’s disease, *FDG PET* fluorodeoxyglucose positron emission tomography, *MRI* magnetic resonance imaging, *PET* positron emission tomography

Studies have shown that biomarkers, including those accessible in plasma, can predict cognitive decline and dementia with high accuracy [97, 98]. As more trials include these biomarkers and more data accrue, it will be possible to understand the trajectory of biomarker change, the relationship of treatment response to baseline levels, and the magnitude of change in biomarkers that correlates with meaningful benefit on clinical outcomes. These data may guide the identification of additional, validated surrogate biomarker endpoints for future AD trials and eventual clinical monitoring [99]. New information regarding the relationship of specific biomarkers to drug mechanism of action will be key to choosing the most appropriate biomarkers for trials and using them to interpret treatment effects. It is anticipated that some biomarker effects will be predominantly class-based (e.g., anti-amyloid monoclonal antibodies will best be assessed by their ability to lower amyloid plaque levels, which may be less important for a future anti-inflammatory DMT), while others may be more mechanism-independent (e.g., neurofilament light chain holds promise as a marker of downstream neurodegeneration) regardless of the cause of the neuronal death [100]. In addition, a profile, ratio, or composite of biomarkers may be more informative than single biomarker measures.

## Next steps and gaps: moving meaningful benefits forward

The AD field is undergoing a period of dramatic and rapid transition. The emergence of disease-modifying therapies in AD is reasonably associated with more questions than answers at present. Accordingly, we offer our proposal as a *first step* in a necessary discussion the field must entertain as pivotal data about the utility of DMTs continues to emerge. Much is not yet known: it is anticipated that there will soon be answers as to whether the removal of amyloid plaque is truly an acceptable surrogate marker; this, in turn, may enable the registration of multiple drugs. Relationships between amyloid removal and shorter-term cognitive/functional benefit will also be better understood.

Other questions will require creativity and additional data sets to address. For example, how long do we need to follow up with participants from pivotal clinical trials to confirm predictive benefits from measures demonstrated in these trials? The answer to that question must consider issues of replicability in other trials, as well as pragmatics (e.g., some placebo-controlled studies cannot ethically be extended for many years), and how much real-world and open-label data can be reasonably compiled and analyzed to provide answers both cross-sectionally and longitudinally.

The challenges the field of AD faces are not unique to this disease area—for example, there are currently many FDA-approved drugs for multiple sclerosis with different mechanisms of action that target distinct immune-mediated disease processes [101]. A framework similar to biomarker-guided treatment development and use similar to that of multiple sclerosis may guide drug development efforts in AD. Personalized care and treatment of AD based upon a precision medicine approach that takes into account individual differences in biology, lifestyle, and environment and aims to optimize the effectiveness of disease prevention and treatment is the goal of AD drug development and clinical care. Implementation of the meaningful benefit approach will facilitate accomplishing this goal by looking at the data through different lenses.

## Conclusions

DMTs have the potential to transform AD treatment paradigms. Translating clinical trial results into clinical practice will be crucial in demonstrating the anticipated meaningful benefits of DMTs to the diverse stakeholders of the AD community. Healthcare providers, patients and their caregivers, regulators, and payers apply different metrics and thresholds when considering the efficacy of a drug and the meaningfulness of its effects. Demonstrating meaningful benefits in AD in a robust, multidimensional way may enable physicians to communicate the potential outcomes of therapy to their patients and caregivers and track progress on an individual level. The *meaningful benefits* framework discussed in this paper may serve to facilitate such a goal. This novel approach is particularly important as drug development in AD moves into the earlier stages of disease and can help bridge the gap between the emergence of a DMT and understanding its clinical application. A more comprehensive understanding of the data, communicated consistently using this proposed multidimensional model, will facilitate decision-making among AD stakeholders and establish reasonable expectations regarding the use of DMTs in the future.

## Data Availability

Not applicable.

## Abbreviations

A4: Anti-Amyloid Treatment in Asymptomatic Alzheimer’s study
AD: Alzheimer’s disease
ADAS-Cog: Alzheimer’s Disease Assessment Scale, Cognitive Subscale
ADCOMS: Alzheimer’s Disease Composite Score
ADCS ADL-PI: Alzheimer’s Disease Cooperative Study—Activities of Daily Living Prevention Instrument
ADCS-iADL: Alzheimer's Disease Cooperative Study—Instrumental Activities of Daily Living
APCC: Alzheimer’s Prevention Initiative Composite Cognitive Test
API: Alzheimer’s Prevention Initiative
CDR-GS: Clinical Dementia Rating – Global Score
CDR-SB: Clinical Dementia Rating – Sum of Boxes
CFI: Cognitive Function Index
COA: Clinical outcomes assessment
DMT: Disease-modifying therapy
ECog: Everyday Cognition scale
EMA: European Medicines Agency
FDA: US Food and Drug Administration
FDG PET: Fluorodeoxyglucose positron emission tomography
iADRS: Integrated Alzheimer’s Disease Rating Scale
MBI: Mild behavioral impairment
MCI: Mild cognitive impairment
MCID: Minimum clinically important difference
MMSE: Mini-Mental State Examination
MRI: Magnetic resonance imaging
NIA-AA: National Institute on Aging and Alzheimer’s Association
NNH: Number needed to harm
NNT: Number needed to treat
NTB: Neuropsychological Test Battery
OR: Odds ratio
PACC: Preclinical Alzheimer Cognitive Composite
PET: Positron emission tomography
PRO: Patient-reported outcome
RR: Relative risk
TTE: Time to event
UPSA: University of California San Diego Performance-Based Skills Assessment
VRFCAT: Virtual Reality Functional Capacity Assessment Tool
